# Primary adult choroid plexus carcinomas: a single-center experience with a systematic review

**DOI:** 10.3389/fonc.2023.1260116

**Published:** 2023-10-31

**Authors:** Pengcheng Zuo, Yiying Mai, Zhuang Jiang, Bochao Zhang, Yujin Wang, Mingxin Zhang, Zhen Wu, Junting Zhang, Liwei Zhang

**Affiliations:** ^1^ Department of Neurosurgery, Beijing Tiantan Hospital, Capital Medical University, Beijing, China; ^2^ China National Clinical Research Center for Neurological Diseases, Beijing, China

**Keywords:** primary, adult, choroid plexus carcinoma, gross-total resection, radiotherapy

## Abstract

**Objective:**

Primary adult choroid plexus carcinomas (PACPCs) are extremely rare brain tumors. The existing literature primarily comprises case reports, which limits our understanding of this uncommon disease. This study aims to describe the clinical characteristics and prognosis of PACPCs, as well as to identify optimal treatment strategies.

**Methods:**

We conducted a comprehensive analysis of clinical data from 7 patients with PACPCs who underwent surgical treatment at the Department of Neurosurgery, Beijing Tiantan Hospital, between March 2011 and March 2023. Additionally, a thorough search of the PubMed database was performed using the keywords “choroid plexus carcinoma” or “choroid plexus carcinomas” within the time frame of August 1975 to April 2023, which yielded a total of 28 identified cases. Subsequently, we evaluated risk factors for progression-free survival (PFS) and overall survival (OS) based on the pooled cases.

**Results:**

The pooled cohort, consisting of 7 cases from our institution and 28 cases from the literature, included 20 males and 15 females with a mean age of 44.3 ± 14.7 years (range: 21-73 years). Gross-total resection (GTR) and non-GTR were achieved in 22 (62.9%) and 13 (37.1%) patients, respectively. Radiotherapy and chemotherapy were administered to 29 (90.6%) and 13 (40.6%) patients, respectively. After a mean follow-up of 21.0 ± 26.7 months (range: 2-132 months), 18 patients were alive, and 11 patients had died. The multivariate Cox regression model demonstrated that non-GTR (HR 5.262, 95% CI 1.350-20.516, p=0.017) was a negative prognostic factor for OS. However, we did not find any risk factors for PFS.

**Conclusion:**

Complete surgical resection should be considered as the primary treatment approach for this rare disease. Chemotherapy and radiotherapy appear to have limited effectiveness in treating this condition. Further research with large cohorts is needed to validate our conclusions.

## Introduction

Choroid plexus tumors (CPTs) are extremely rare, accounting for only 0.3%-0.6% of brain tumors (1). Choroid plexus carcinomas (CPCs), classified as Grade III tumors by the World Health Organization, represent the most aggressive form of CPTs. They exhibit distinct malignant features, such as a high number of mitotic figures, dense cellularity, loss of clear papillary growth pattern, presence of necrosis, and infiltration into the surrounding brain tissue (2). It primarily occurs in children and rarely affects adults (3). An increasing number of studies have delved into the research of pediatric CPCs (4, 5). However, due to the rarity of adult CPCs, there is a scarcity of literature that systematically investigates the rare disease. In light of this, we have collected cases of primary adult choroid plexus carcinomas (PACPCs) from our institution and extensively gathered reported cases from the literature, aiming to describe the clinical characteristics and prognosis of this rare disease and provide the current optimal treatment approach.

## Methods

We conducted a retrospective analysis of 12 cases of PACPCs. All patients underwent surgery at Beijing Tiantan Hospital between March 2011 and March 2023. However, 4 patients were lost to follow-up, and 1 patient was diagnosed with secondary choroid plexus carcinoma associated with lung cancer. After excluding these 5 patients, a total of 7 cases were included in our study. Comprehensive systemic examinations ruled out potential additional tumors in these 7 patients. The collected clinical data consisted of age, sex, tumor location, extent of tumor resection, histopathological examinations, treatment protocol, and clinical outcomes. Pre- and postoperative MRI images were used to assess the extent of tumor excision, categorized as gross total resection (GTR) or non-GTR. Follow-up was conducted through telephone interviews. The pathological diagnosis of PACPCs was confirmed by the Department of Neuropathology at Beijing Neurosurgical Institute, following the 2021 World Health Organization Classification of Tumors of the Central Nervous System. To perform a pooled analysis of PACPCs, we conducted a search in the PubMed database from August 1975 to April 2023 using the keywords “choroid plexus carcinoma” or “choroid plexus carcinomas,” resulting in the inclusion of a total of 28 cases. Cox regression models were employed to evaluate variables and their association with progression-free survival (PFS) and overall survival (OS). The Kaplan-Meier method was used to determine differences in OS and PFS, with p-values calculated using the log-rank test. Statistical analyses were conducted using the SPSS Statistical Package software, with a significance level set at p < 0.05. It’s worth noting that the pooled cases with incomplete data were subsequently excluded from our analysis. Consequently, our final analysis included 29 cases with complete data for assessing risk factors related to OS and 25 cases for analyzing risk factors related to PFS.

## Results

### Clinical characteristics

The pooled cohort (including 7 cases from our institute and 28 cases from the literature) included 20 (57.1%) males and 15 (42.9%) females with a mean age of 44.3 ± 14.7 years (range: 21-73 years). The lesion locations included lateral ventricle (n=20), fourth ventricle (n=7), third ventricle (n=2), multiple lesions (n=2), cerebellopontine angle (n=1), occipital lobe (n=1), temporal lobe (n=1) and temporoparietal lobe (n=1). All patients’ lesions showed evident enhancement on imaging examinations (32 cases available). Gross-total resection (GTR) and non-GTR were achieved in 22 (62.9%) and 13 (37.1%) patients, respectively. Radiotherapy and chemotherapy were administered to 29 (90.6%) and 13 (40.6%) patients, respectively (**Table 1**). 7 (25%) cases presented with tumor dissemination or metastasis. The summary data of the cohort was presented in **Table 2**.

**Table 1:** Clinical data of ACPCs from literature and our institute.

Case	Sex	Age (years)	Location	Enhancement	Extent of resection	RT	CT	Recurrence	Therapy after recurrence	Metastasis	Outcome	PFS (months)	FU (months)
Cases from literature (28 cases)													
Witten et al, 2021 (3)	F	33	Cerebellopontine Angle	Yes	Non-GTR	NA	NA	NA	NA	NA	NA	NA	NA
Jo et al, 2021 (6)	M	46	Multiple	Yes	Non-GTR	Yes	No	No	–	Yes	Dead	4	4
Crea et al, 2020 (7)	F	38	Third ventricle	Yes	GTR	Yes	No	No	–	No	Alive	6	6
Kim et al, 2019 (8)	M	40	Fourth ventricle	Yes	Non-GTR	Yes	Yes	NA	NA	Yes	Dead	0	11
	F	49	Lateral ventricle	Yes	GTR	No	Yes	No	–	No	Alive	10	10
Pellerino et al, 2014 (9)	M	50	Fourth ventricle	Yes	GTR	Yes	Yes	Yes	RT+CT	Yes	Alive	84	132
Ozdogan et al, 2015 (10)	M	73	Fourth ventricle	Yes	GTR	NA	NA	NA	NA	NA	NA	NA	NA
Guo et al, 2015 (11)	M	59	Temporoparietal lobe	Yes	GTR	Yes	Yes	Yes	S	NA	Alive	6	6
Bohara et al, 2015 (12)	M	60	Lateral ventricle	Yes	Non-GTR	Yes	Yes	NA	–	NA	Dead	NA	13
Yip et al, 2014 (13)	M	21	Lateral ventricle	Yes	GTR	Yes	Yes	No	–	No	Alive	24	24
Kishore et al, 2012 (14)	M	24	Lateral ventricle	Yes	Non-GTR	Yes	No	NA	NA	NA	NA	NA	NA
Misaki et al, 2011 (15)	M	38	Lateral ventricle	Yes	Non-GTR	Yes	Yes	Yes	RT+CT	No	Dead	3	11
Lozier et al, 2009 (16)	F	68	Temporal lobe	Yes	GTR	Yes	Yes	No	–	No	Alive	44	44
Fernández Calvo et al, 2007 (17)	M	26	Occipital lobe	Yes	GTR	Yes	No	Yes	RT+CT	Yes	Alive	33	33
Osada et al, 2006 (18)	M	53	Lateral ventricle	Yes	Non-GTR	Yes	No	No	–	No	Alive	7	7
Han et al, 2006 (19)	F	35	Lateral ventricle	Yes	GTR	Yes	No	No	No	No	Alive	8	8
Guan et al, 2006 (20)	M	52	Lateral ventricle	NA	GTR	NA	NA	NA	NA	NA	NA	NA	NA
Fabi et al, 2005 (21)	F	58	Multiple	Yes	Non-GTR	Yes	No	Yes	CT	No	Dead	35	38
İZCİ et al, 2005 (22)	M	54	Lateral ventricle	Yes	GTR	Yes	No	NA	–	NA	Dead	NA	NA
Wyatt et al, 2001 (23)	M	32	Lateral ventricle	Yes	Non-GTR	Yes	No	Yes	S	No	Dead	13	48
Kohmura et al, 2000 (24)	F	49	Lateral ventricle	Yes	Non-GTR	Yes	No	No	–	No	Alive	NA	NA
Hashizume et al, 1995 (25)	M	68	Lateral ventricle	Yes	Non-GTR	Yes	No	No	–	No	Alive	2	2
Başkaya et al, 1994 (26)	M	22	Lateral ventricle	Yes	GTR	Yes	No	No	–	No	Alive	8	8
Matsuda et al, 1991 (27)	F	31	Lateral ventricle	Yes	GTR	Yes	No	No	–	No	Alive	14	14
Itoh et al, 1986 (28)	F	25	Fourth ventricle	Yes	GTR	Yes	No	Yes	No	Yes	Dead	2	7
Carpenter et al, 1982 (29)	F	29	Lateral ventricle	Yes	GTR	No	No	No	–	No	Alive	12	12
Vaquero et al, 1979 (30)	F	26	Lateral ventricle	NA	GTR	Yes	No	Yes	S+RT	Yes	Alive	48	52
Dohrmann et al, 1975 (31)	F	55	Fourth ventricle	NA	Non-GTR	Yes	No	No	–	No	Alive	4	4
Cases from literature (7 cases)													
Case 1	F	39	Lateral ventricle	Yes	GTR	Yes	No	Yes	No	No	Dead	6	7
Case 2	M	51	Lateral ventricle	Yes	Non-GTR	Yes	Yes	Yes	No	Yes	Dead	NA	5
Case 3	F	47	Fourth ventricle	Yes	GTR	Yes	Yes	No	–	No	Alive	58	58
Case 4	F	60	Third ventricle	Yes	GTR	Yes	Yes	No	–	No	Dead	12	12
Case 5	M	66	Fourth ventricle	Yes	GTR	No	No	NA	NA	No	Dead	12	12
Case 6	M	40	Lateral ventricle	Yes	GTR	Yes	Yes	Yes	No	No	Dead	2	4
Case 7	M	34	Lateral ventricle	Yes	GTR	Yes	Yes	No	–	No	Alive	17	17

F, female; M, male; NA, not available; GTR, gross total resection; RT, radiotherapy; CT, chemotherapy; PFS, progression-free survival; FU: follow up.

**Table 2:** Summary of clinical characteristics of ACPCs.

Variable	Value
Overall	35
Sex	
Male	20 (57.1%)
Female	15 (42.9%)
Age (years)	44.3 ± 14.7
Location	
Lateral ventricle	20 (57.1%)
Others	15 (42.9%)
Extent of resection	
GTR	22 (62.9%)
Non-GTR	13 (37.1%)
RT	
Yes	29 (90.6%)
No	3 (9.4%)
CT	
Yes	13 (40.6%)
No	19 (59.4%)
Enhancement	32 (100%)
Outcome	29 available
Dead	11 (37.9%)
Alive	18 (62.1%)
Mean FU	21.0 ± 26.7 months
Recurrence	25 available
Yes	10 (40%)
No	15 (60%)
Mean PFS	18.5 ± 20.8 months

### Pooled analysis

**Figure 1:**
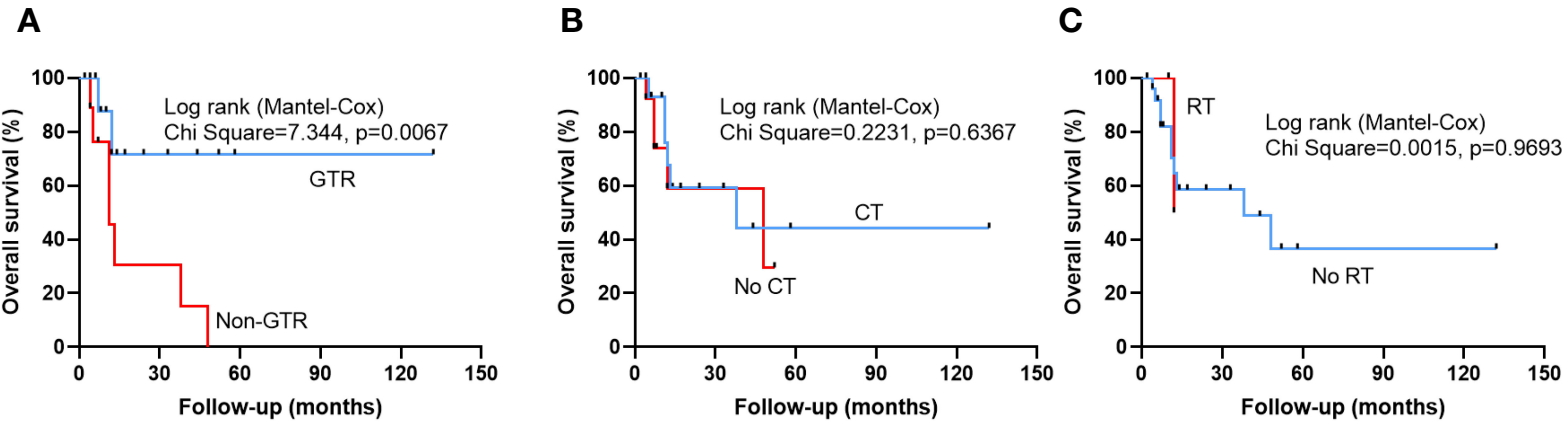
Kaplan-Meier survival curves illustrating patients who underwent gross total resection (GTR) exhibited a significantly improved OS compared to Non-GTR group **(A)**. However, there was no statistically significant difference between patients who received chemotherapy (CT) and patients who did not receive CT **(B)**, and patients who received radiotherapy (RT) and patients who did not receive RT **(C)**.

After a mean follow-up of 21.0 ± 26.7 months (range: 2-132 months), 18 patients were alive and 11 patients were dead. The univariate cox regression analysis revealed that non-GTR (HR 4.611, 95% CI 1.342-15.839, p=0.015) was a only risk factor to predict a poor OS (**Table 3**). Multivariate cox regression analysis confirmed that non-GTR (HR 5.262,95% CI 1.350-20.516, p=0.017) was the independent risk factor in OS (**Table 3**). Kaplan-Meier analysis also showed that non-GTR (p=0.0067) predicted a poor OS (**Figures 1A**), Median OS of non-GTR was 11 months, approximately 70% of patients who underwent complete surgical resection survived We did not observe any significant effect of radiotherapy or chemotherapy in prolonging the OS in this disease (**Figures 1B, C**). However, Median Survival Time of CT and no CT was 38 months and 48 months, respectively.

**Table 3:** Cox regression model for risk factors predicting OS.

Variable	Number of patients	Dead (%)	Univariate Analysis		Multivariate Analysis	
			HR (95% CI)	P value	HR (95% CI)	P value
Overall	29	11 (37.9%)				
Sex						
Male	16	7 (43.8%)	Reference			
Female	13	4 (30.8%)	0.579 (0.168-1.997)	0.387		
Age (years)						
<40	13	4 (30.8%)	Reference			
≥40	16	7 (43.8%)	1.732 (0.504-5.956)	0.383		
Location						
Lateral ventricle	16	6 (37.5%)	Reference			
Others	13	6 (46.2%)	1.067 (0.341-3.340)	0.911		
Extent of resection						
GTR	19	4 (21.1%)	Reference		Reference	
Non-GTR	10	7 (70%)	4.611 (1.342-15.839)	0.015*	5.262 (1.350-20.516)	0.017*
RT					
Yes	26	10 (38.5%)	Reference		Reference	
No	3	1 (33.3%)	0.961 (0.118-7.818)	0.970	2.011 (0.179-22.524)	0.571
CT						
Yes	15	6 (40%)	Reference		Reference	
No	14	5 (35.7%)	1.328 (0.399-4.414)	0.644	1.219 (0.337-4.412)	0.762

*: p<0.05 was considered statistical significant.

**Figure 2:**
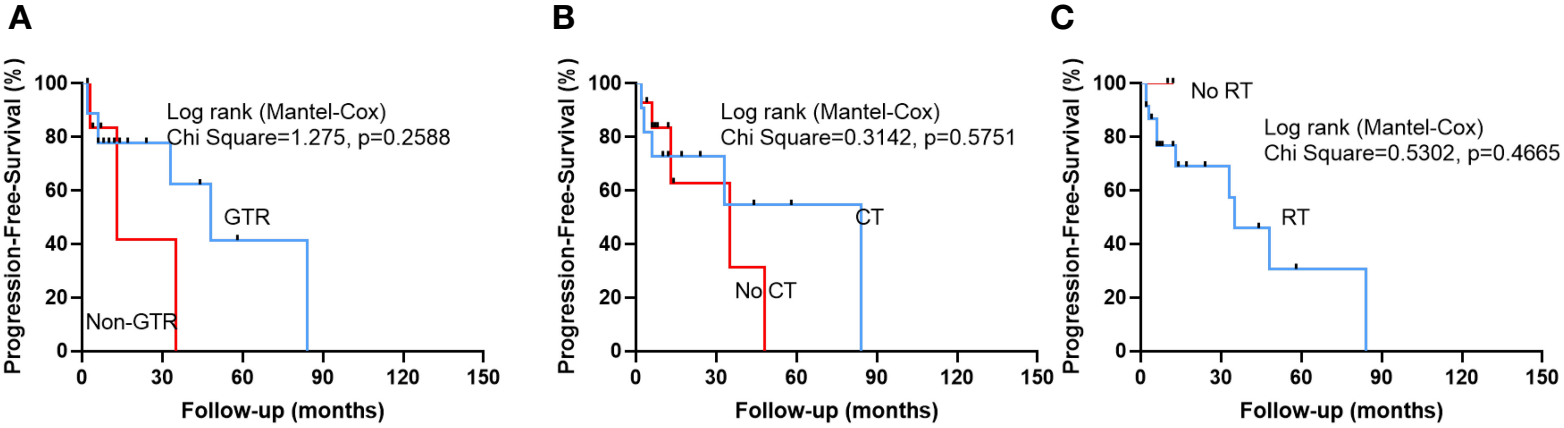
Kaplan-Meier survival curves show that there was no statistically significant difference between patients who achieved GTR and patients who did not achieve GTR, and patients who received CT and patients who did not receive CT **(B)**, and patients who received radiotherapy (RT) and patients who did not receive RT.

After a median follow-up of 18.5 ± 20.8 months (range: 2-84 months), 10 (40%) patients suffered tumor recurrence. However, we did not identify any significant risk factors that influence the PFS of this disease (**Table 4** and **Figure 2**). However, Median PFS of GTR and non-GTR was 48 VS 13 months, respectively. Median PFS of CT and no CT was 84 months VS 35 months, respectively.

**Table 4:** Cox regression model for risk factors predicting PFS.

Variable	Number of patients	Recurrence (%)	Univariate Analysis		Multivariate Analysis	
			HR (95% CI)	P value	HR (95% CI)	P value
Overall	25	10 (40%)				
Sex						
Male	12	6 (50%)	Reference			
Female	13	4 (30.8%)	0.526 (0.137-2.014)	0.348		
Age (years)						
<40	13	6 (46.2%)	Reference			
≥40	12	4 (33.3%)	0.410 (0.097-1.738)	0.226		
Location						
Lateral ventricle	14	5 (35.7%)	Reference			
Others	11	5 (45.5%)	0.613 (0.151-2.483)	0.493		
Extent of resection						
GTR	18	7 (38.9%)	Reference		Reference	
Non-GTR	7	3 (42.9%)	2.232 (0.525-9.487)	0.277	1.966 (0.360-10.738)	0.435
RT						
Yes	23	10 (43.5%)	Reference		Reference	
No	2	0 (0%)	0.043 (0.000-26289.330)	0.643		0.990
CT						
Yes	11	5 (45.5%)	Reference		Reference	
No	14	5 (35.7%)	1.457 (0.382-5.552)	0.581	1.085 (0.225-5.228)	0.919

*: p<0.05 was considered statistical significant.

### Illustrative case

#### Case 4

**Figure 3:**
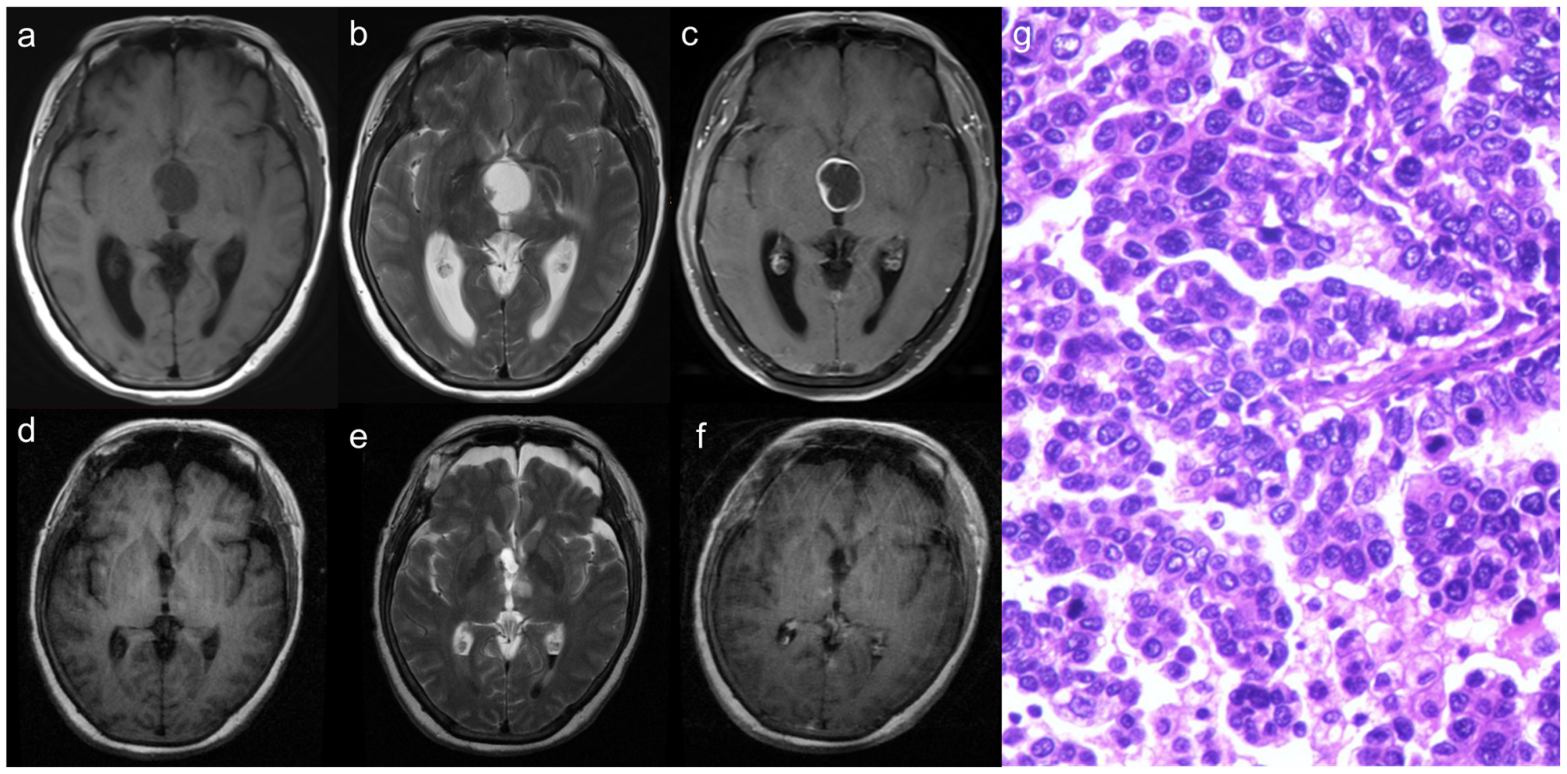
The solid part of the tumor demonstrates isointense on both T1 and T2-weighted images **(A, B)**, with significant enhancement observed following contrast agent injection **(C)**. After surgery, the tumor was resected totally **(D–F)**. Histopathology demonstrated that a significant presence of pleomorphic tumor cells with a blurring of papillary architecture **(G)**.

A 60-year-old female patient presented with a complaint of headaches persisting for the past 5 months and was admitted to the Department of Neurosurgery at Beijing Tiantan Hospital. A preoperative cranial MRI revealed a cystic-solid mass located within the third ventricle. The cystic wall and the solid portion of the mass showed isointense on both T1- and T2-weighted images. Following the administration of a contrast agent, significant enhancement was observed (**Figures 3A–C**). Neurological examination indicated a decline in bilateral visual acuity. The transfrontal-transventricular approach was employed, and the tumor was completely excised (**Figures 3D–F**). Postoperative pathology revealed a significant presence of pleomorphic tumor cells with a blurring of papillary architecture (**Figure 3G**) and the immunohistochemistry results showed a Ki-67 index of 50%. After discharge, the patient received both radiotherapy and chemotherapy. However, during subsequent follow-up, the patient, unfortunately, died 12 months after the procedure.

## Discussion

Choroid plexus carcinoma (CPC) is extremely rare and is predominantly found in children, occurring rarely in adults (32). Due to its rarity, there is currently a lack of data on the incidence of this condition. From 2011 to 2023, our center treated a total of approximately 500 patients with Choroid plexus tumors (CPTs), of which 68 cases were diagnosed as CPCs, accounting for approximately 13.6% of all CPTs. Among the 68 cases of CPCs, 12 cases were adults, representing approximately 2.4% of all 500 cases.

The histopathological diagnosis of CPC is featured increased cell density, increased mitotic figures (usually≥5 per 10 high- power fields) and necrosis (33). While choroid plexus papillomas have distinct papillary structures, the papillary features in CPC are often not evident or even lost (34). CPC primarily needs to be differentiated from three other types of intraventricular brain tumors. First are the choroid plexus papilloma. Both of these tumors exhibit distinct papillary structures, have a lower proliferation index (Ki-67, range:0.98%-2.22%) (35), while CPC lacks clear papillary features and often has a higher proliferation index (Ki-67≥50%) (36). The second type of tumor requiring differentiation is ependymoma, particularly the papillary variant. It can be distinguished by its characteristic cytological and morphological features on histological examination. These features include stippled chromatin, ependymal rosettes (37). The third type is papillary meningioma, a rare high-grade (WHO grade III) variant of meningioma that can occur in the choroid plexus. This tumor is histologically described as having a pseudopapillary or perivascular pattern (38), which distinguishes it from CPC. The results of immunohistochemical studies of CPC exhibit considerable variability. Gottschalk (39) et al. reported varying degrees of positive immunoreactions in CPP or CPC for several markers, including cytokeratin, S-100 protein, vimentin, epithelial membrane antigen, and others. Notably, Inamura (40) et al. and Osada et al (18) both found that the levels of CA19-9 decreased rapidly after the removal of the tumor. CA19-9 shows promise in detecting the recurrence of CPC. We found that 25% of the cases exhibited tumor dissemination in the cerebrospinal fluid or even distant metastases, indicating that adult CPC demonstrates a more malignant biological behavior.

Surgery is the primary approach for treating pediatric CPCs. Sun et al. conducted an analysis of clinical data from 102 pediatric patients (age ≤ 18 years) with CPCs and found that complete surgical resection significantly improves the OS of these patients (4). In our study, complete surgical resection was also found to be advantageous in improving OS of PACPCs. Furthermore, complete surgical resection improved the median PFS of these patients. However, it’s important to note that GTR did not exhibit a significant statistical difference in improving PFS. Based on these findings, if feasible, efforts should be made to achieve complete surgical resection in this rare disease.

Chemotherapy plays an important role in pediatric CPCs. A previous study conducted a statistical analysis of 135 cases of pediatric CPCs to explore the role of radiotherapy and chemotherapy. The authors found that chemotherapy alone or in combination with radiotherapy contributed to improving OS. However, radiotherapy alone did not improve OS (5). Another study showed that chemotherapy play a significant role in achieving long-term survival, but unfortunately, it cannot completely prevent the occurrence of recurrences (41). In our study, there was no significant statistical difference observed in the OS and PFS between patients who received chemotherapy and those who did not. Despite the lack of statistical difference, we found that the median PFS of patients who received chemotherapy was higher than that of patients who did not receive chemotherapy. This finding may suggest a potential role of chemotherapy in CPC. Regarding radiotherapy, due to the fact that the majority of patients (90.6%) in our cases received radiotherapy, we cannot accurately determine the role played by radiotherapy in this disease.

Overall, adult CPC has a poor prognosis, and complete surgical resection is currently the most effective approach to improve patient OS. The roles of radiotherapy and chemotherapy require further validation through more cases.

## Conclusion

Complete surgical resection should be considered as the primary treatment approach for this rare disease. Chemotherapy appears to have limited effectiveness in treating this condition. Due to the fact that the majority of patients underwent radiation therapy, we cannot assess the specific impact of radiotherapy on the disease. Further research with large cohorts is needed to validate our conclusions.

## Data availability statement

The original contributions presented in the study are included in the article/supplementary material. Further inquiries can be directed to the corresponding author.

## Ethics statement

The studies involving humans were approved by human research ethics committee of Beijing Tiantan Hospital. The studies were conducted in accordance with the local legislation and institutional requirements. The participants provided their written informed consent to participate in this study. Written informed consent was obtained from the individual(s) for the publication of any potentially identifiable images or data included in this article.

## Author contributions

PZ: Writing – original draft. YM: Formal Analysis, Writing – original draft. ZJ: Formal Analysis, Writing – original draft. BZ: Data curation, Writing – original draft. YW: Investigation, Writing – original draft. MZ: Investigation, Writing – original draft. ZW: Resources, Writing – review & editing. JZ: Resources, Writing – review & editing. LZ: Writing – review & editing.
